# Novel Candidate Microorganisms for Fermentation Technology: From Potential Benefits to Safety Issues

**DOI:** 10.3390/foods11193074

**Published:** 2022-10-04

**Authors:** Duygu Ağagündüz, Birsen Yılmaz, Tevfik Koçak, Hilal Betül Altıntaş Başar, João Miguel Rocha, Fatih Özoğul

**Affiliations:** 1 Department of Nutrition and Dietetics, Gazi University, Emek, Ankara 06490, Turkey; 2 Department of Nutrition and Dietetics, Cukurova University, Sarıcam, Adana 01380, Turkey; 3 Laboratory for Process Engineering, Environment, Biotechnology and Energy, Faculty of Engineering, University of Porto, 4050-345 Porto, Portugal; 4 Associate Laboratory in Chemical Engineering, Faculty of Engineering, University of Porto, 4050-345 Porto, Portugal; 5 Department of Seafood Processing Technology, Faculty of Fisheries, Cukurova University, Balcali, Adana 01330, Turkey

**Keywords:** novel microorganisms, fermentation, function, safety, health

## Abstract

Fermentation is one of the oldest known production processes and the most technologically valuable in terms of the food industry. In recent years, increasing nutrition and health awareness has also changed what is expected from fermentation technology, and the production of healthier foods has started to come a little more forward rather than increasing the shelf life and organoleptic properties of foods. Therefore, in addition to traditional microorganisms, a new generation of (novel) microorganisms has been discovered and research has shifted to this point. Novel microorganisms are known as either newly isolated genera and species from natural sources or bacterial strains derived from existing bacteria. Although novel microorganisms are mostly studied for their use in novel food production in terms of gut-microbiota modulation, recent innovative food research highlights their fermentative effects and usability, especially in food modifications. Herein, *Clostridium butyricum*, *Bacteroides xylanisolvens*, *Akkermansia muciniphila*, *Mycobacterium setense manresensis*, and *Fructophilic lactic acid bacteria* (*FLAB*) can play key roles in future candidate microorganisms for fermentation technology in foods. However, there is also some confusion about the safety issues related to the use of these novel microorganisms. This review paper focuses on certain novel candidate microorganisms for fermentation technology with a deep view of their functions, benefits, and safety issues.

## 1. Introduction

One of the ancient methods of food processing, fermentation is responsible for a significant portion of the food consumed by humans [1]. The oldest fermented foods for which archaeological evidence is accessible are cereal products, bread, and beer [2,3]. Around 14,000 years ago, fermented products accompanied and probably aided the shift from hunter-gatherer groups to sessile farming communities. Since then, these foods have been a constant in human diets and especially recently, their popularity has been increasing as the demand for consuming functional foods has substantially expanded [4,5]. However, it is still possible to find different definitions of fermentation and fermented food.

The International Scientific Association for Probiotics and Prebiotics (ISAPP) consensus has defined fermented foods as “foods made through desired microbial growth and enzymatic conversion of food components” [5]. The definition of fermented foods not only covers foods that contain live microorganisms at the time of consumption but also includes fermented foods such as heat-treated or pasteurized fermented foods and bread, which might not contain live microorganisms during consumption [5].

Fermentation can be classified in different ways based on the microorganisms used in the production, the main metabolites produced by these microorganisms, the type of food used as the starting material, mode of cultivation, oxygen need, water activity and nutrient metabolism [6,7]. According to the microorganisms involved in the fermentation and the end product, the most common categories of fermentation are lactic acid (dairy, vegetable, cereal, and meat), acetic acid (vinegar), ethanol/alcohol (baking, brewing, and winemaking), and alkaline (Japanese natto) [6,7]. Lactic acid bacteria (LAB), primarily species of *Enterococcus*, *Leuconostoc*, *Lactobacillus*, *Pediococcus*, *Lactococcus*, and *Weissella*, are commonly found in many fermented products, while some novel microorganisms have recently been used in food fermentation [8,9]. LAB are the predominant microbiota of all identified fermented foods and beverages, and it has been thought that they are the most significantly contributors to the health benefits of these foods and beverages [10]. LAB-driven fermentation frequently results in by-products with bioactivity and various health-promoting properties, including immunomodulatory, antiallergenic, antiobesity, antioxidant activities as well as increasing the bioavailability of vitamins and minerals in foods [11]. Several functional properties of microorganisms used in fermentation have been reported. The selection of starter cultures to be used in the food industry should consider factors such as fibrinolytic activity, poly-glutamic acid, antinutritional compound degradation, probiotic, antibacterial, and antioxidant capabilities [9]. Moreover, it has been stated that microorganisms used in food fermentation can enhance the safety (foodborne pathogens) and shelf life of fermented foods thanks to their probiotic properties [12]. These favorable effects of microorganisms were traditionally the main goal of the development of fermented foods and the fermentation process. Along with these properties, fermentation may support the substrate’s biochemical transformations, enhancing the nutritional content of foods and improving the organoleptic properties of foods [13].

The rapid increase in the potential health effects and functional properties of fermented foods and microorganisms used in fermentation has brought with it some global regulatory issues [13]. A motivation for fermenting foods, especially dairy products, vegetables, fish, and fermented meats, is to preserve foods and increase their safety [1]. Even though there is a common global regulation for fermented foods worldwide, in the European Union countries it is the General Food Law Regulation (Regulation (EC) No 178/2002; current consolidated version 26 July 2019). The aim of this regulation is “to ensure a high level of protection of human life and consumer interests concerning food while ensuring the effective functioning of the internal market” [14]. The potential health benefits of fermented foods and traditional microorganisms involved in fermentation have been shown in many studies [15,16,17]. Fermented foods are accepted as safe for intended use according to the ISAPP consensus; however, novel fermented foods and microorganisms which have recently been used in fermentation need to be well understood through preclinical and clinical studies [7].

This study reviews some novel candidate microorganisms for food fermentation technology with a deep view of their functions, benefits, and safety issues.

## 2. Certain Novel Microorganisms for Fermentation

Fermentation is one of the oldest biological food preservation methods, mainly known for enhancing the organoleptic properties of foods in the desired direction and extending food shelf life [18,19]. The nutritional value of the food increases as essential amino acids, vitamins, and antimicrobial metabolites such as bacteriocin are synthesized during fermentation, which also breaks down fermentable carbohydrates into organic acids, carbon dioxide, and alcohol [20]. These antimicrobial metabolites contribute to food safety by preventing the spoilage of foods by microorganisms [21]. Moreover, fermentation reduces antinutritional factors, increases the digestibility of foods, and ultimately improves the sensory properties of foods [20].

Fermentation can be categorized according to the relevant microorganisms (lactic acid, propionic acid, acetic acid, alcohol, carbon dioxide) and primary metabolites, or they can be defined based on food substrates (meat and fish, dairy products, vegetables) [18]. There are three main functional components of fermented foods, namely viable and nonviable probiotics, prebiotics, and biogenic [22]. Antimicrobial, antioxidant (milk, cereals, fruits, vegetables, meat, and fish), antihypertensive (fermented milk products and cereals), antidiabetic, fermentable oligosaccharides, disaccharides, monosaccharides and polyols (FODMAP)-reducing activities of components in fermented foods have been demonstrated, while some microorganisms isolated from fermented foods show probiotic properties [23]. Despite numerous favorable properties of fermentation and microorganisms which are traditionally used in food fermentation, there are still issues with characterization and application that need to be overcome. The characterization procedure of microorganisms has several stages, including exact identification, potential health benefits, and safety assessment. Each of these steps varies depending on the strain, so they must be precisely identified [24].

New-generation genetic modification technologies can be applied to increase the functionality of microorganisms including novel ones for fermentation and benefits. Actually, it is not a new concept to use genetically modified microorganisms for preventative and medicinal purposes. Even though CRISP/Cas9 zinc-finger nucleases, transcription activator-like effector nucleases and other gene editing technologies were not yet available, such concepts were contested in the early 1990s [25]. One of the often-used species for fermentative hydrogen production is *C. butyricum*. To be able to increase the yield of H_2_, some researchers tried genetic manipulations which included the elimination of competing pathways such as the butyrate formation pathway [26,27]. Cai et al. (2011) reported that the *hbd*-deficient strain produced more H_2_ while simultaneously producing less ethanol under low partial pressures of H_2_, suggesting that *C. butyricum*’s synthesis of H_2_ and the route for ethanol formation may compete for NADH [27]. Another study that aimed to demonstrate a genetic approach for increasing H_2_’s yield of *C. butyricum* showed that via using a ClosTron plasmid, it was possible to stop the aldehyde-alcohol dehydrogenase (aad) enzyme from producing ethanol. The resulting aad-deficient mutant demonstrated about 20% improved performance in H_2_ production with the addition of sodium acetate, despite the fact that the eradication of ethanol formation alone did not increase hydrogen production [26]. *Akkermansia muciniphila* is one of the bacteria which gives promising results via genetic modifications. It was hypothesized that ingested *Akkermansia muciniphila* may be programmed to interact with signals released within its environment and respond to information based on the deployment of other strains utilized in the methods such as the CRISPR-Cas system and the nisin-controlled gene expression system. As a result, it may be used to treat metabolic imbalances and tissue diseases, and to promote surgical healing [28]. In a nutshell, even though some novel strains including traditional and genetically modified ones have promising properties for food fermentation and health, they also need to be studied in detail, particularly regarding their functionality and safety issues. In the light of the current knowledge, this article focuses on some bacterial species from novel microorganisms in the following subsections.

### 2.1. Clostridium butyricum

In 2014, the European Commission approved the use of *Clostridium butyricum* (*C. butyricum*) CBM 588 as a novel food ingredient, and then some companies in the UK focused on its use in food supplements [29]. Although its use is mostly related to its positive effects on lipid metabolism and gastrointestinal microbiome modulation, this bacterium has many promising potentials in industry, environment, and health [30,31].

*C. butyricum* is an important microorganism because of its hydrogen productivity. It has been revealed that different organic nutrient media such as glucose, starch, animal fertilizer, agricultural wastes, food residual, and wastewater can be used as substrates in the fermentation operation. Food residual is considered a large part of its waste, which includes extra carbohydrate ingredients and can be noted as a possible raw material for biohydrogen processing [32,33]. *Clostridia* teams are spore-forming anaerobic bacteria and *Enterobacter* spp. (1 mol H_2_/mol hexose) with a higher yield compared to different fermentative anaerobic bacteria teams such as (2 mol H_2_/mol glucose) H_2_ can use glucose for their production. Among the fermentative hydrogen-producing bacteria, *C. butyricum* has been reported to be one of the high hydrogen-producing microorganisms [34]. In addition to this, *C. butyricum* has a broad spectrum of substrate usage efficiency and has been extensively investigated for hydrogen production from various substrates, including used organic waste. It is stated that it plays a role in protecting the environment and reducing waste [35].

*C. butyricum* is advantageous in food fermentation because it requires mild conditions and is anaerobic and independent of B_12_ Although most bacteria do not have a high tolerance for food because of several components that impede microbial growth, *C. butyricum* does not require food pretreatment, such as melting material, electrodialysis, effective carbon suction, and ion barter [36]. *C. butyricum* NCIMB 8082, *C. butyricum* JKT37, *C. butyricum* CWBI1009, and *C. butyricum* L4 are the strains that play a role in food fermentation [37,38,39,40].

The environmental effects of population increase and expanding economic activity highlight the need to switch from linear business models to resources wise and sustainable business models [33]. Using waste as a source of raw materials in industry to minimize negative environmental effects and process costs is one of the key tenets of the circular economy [41]. In this investigation, Liberato et al. (2021) suggest using crude glycerol and corn soaking liquid as the raw materials for the *C. butyricum* NCIMB 8082 strain to produce 1,3-propanediol, an essential chemical mostly employed in the creation of polymers. After 24 h of fermentation in 65 mL serum, *C. butyricum* NCIMB 8082 could thrive in a culture medium containing only crude glycerol and corn-soaking broth, producing 0.51 g.g^−1^ and 6.56 gL^−1^ of 1,3-propanediol [37].

Many microorganisms such as *Citrobacter*, *Klebsiella*, *Enterobacter*, and *Clostridium* species may convert glycerol to 1,3-propanediol [42,43]. Among these, *C. butyricum* has received the most attention due to its high production, substrate tolerance, and prolificacy [33]. Additionally, *C. butyricum* produces 1,3-propanediol independently of B_12_, which typically necessitates a simpler and cheaper growth medium. Acetic acid, butyric acid, propionic acid, H_2_, and butanol are among the other metabolic byproducts of the *C. butyricum* glycerol fermentation [44].

In the recent investigation by Gupta et al. (2022), it was reported that crude glycerol was fermentatively converted to 1,3-propanediol using the *C. butyricum L4* strain. In this investigation, biogas was used to isolate a brand-new strain of *C. butyricum L4*. Due to its nonpathogenic nature, capacity to grow in various pH and temperature ranges, tolerance to high substrate and product concentrations, the generation of negligible products, and its coenzyme B_12_-independent biotransformation, the *C. butyricum L4* strain was mentioned as a potential candidate for industrial use [40,45].

To solve the current global energy issue, hydrogen gas is recommended as a new sustainable energy source. It has a high energy density of 122–142 kJ/g or 2.75 times more energy per unit weight than conventional hydrocarbon fuel. Due to its high energy content and the fact that it burns to produce water rather than greenhouse gases, hydrogen is a clean alternative energy source [46]. Water, primarily made up of glucose, sucrose, xylose, and fructose, was used as a substrate for biohydrogen generation in a study examining the impact of nano zerovalent iron in the biohydrogen production in the anaerobic fermentation of oil palm leaf juice using *C. butyricum* JKT37. It demonstrated a 1.85-fold rise in hydrogen productivity over the control under optimal circumstances. Butyric, acetic, lactic, and formic acids were the main acidogenic products. The effectiveness of the *C. butyricum* JKT37 strain in producing biohydrogen was demonstrated in that investigation [38].

Another strain, *C. butyricum* CWBI1009, was employed in the fermentation of hydrogen. Because of its strong hydrogen generation activity when digesting various carbon sources, the *C. butyricum* CWBI1009 strain was chosen for the investigation to assess the impact of nitrogen supply on hydrogen production metabolism and hydrogenases (glucose, starch, or disaccharides) [39,47]. Furthermore, the strain worked admirably in immobilized cultures (up to 3.4 mol H_2_/mol glucose) [48]. The *C. butyricum* CWBI1009 strain proved successful in producing hydrogen after the investigation [49].

The Gram-positive endophytic bacterium *C. butyricum* has considerable endurance to the gastrointestinal environment and a separate system of digestive enzymes that provides it with anaerobic probiotic capabilities. It can manufacture various chemicals, including butyric acid, tiny peptides, enzymes, and vitamins that it can use to give the host energy [50]. The growth of digestive epithelium tissue can be aided by butyric acid, which can also strengthen the protective barrier’s ability to function. Volatile fatty acids are said to be crucial for metabolism and intestinal microbiota according to numerous studies [51]. Additionally, additional *C. butyricum* metabolites, such as teichoic acid, encourage *C. butyricum* colonization in the digestive tract because of its strong adhesive qualities [52]. The primary metabolic products of *C. butyricum*, butyric acid, bacteriocin, and enzymes, enhance antioxidant capacity, reduce infection, control intestinal immune function, and maintain healthy gastrointestinal barriers in mice and people [53].

**Figure 1:**
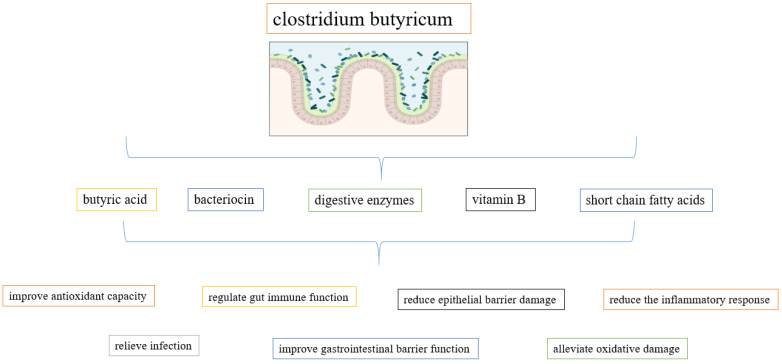
Metabolites and beneficial effects of *C. butyricum*.

*C. butyricum* is a stringent anaerobe that can withstand acids and high temperatures. During its metabolic process, this organism is capable of producing many digestive enzymes, vitamin B, and short-chain fatty acids [54]. According to reports, *C. butyricum* can significantly lessen oxidative damage, inflammation, and damage to the epithelial barrier [54]. The metabolites and beneficial effects produced by *C. butyricum* are shown in Figure 1.

### 2.2. Bacteroides xylanisolvens

In Chassard et al. (2008), *Bacteroides xylanisolvens* was first isolated and described from human feces. According to the analysis of the 16S rRNA gene sequence, the isolates belonged to the genus *Bacteroides* and were linked to one another closely (99.0% sequence similarity) [55]. Some *Bacteroides* species perform advantageous metabolic processes that entail the fermentation of carbohydrates, the use of nitrogenous materials, and the biotransformation of bile acids and other steroids, among other benefits for human health [56]. Keeping intestinal pathogens from populating the intestines concurrently short-chain fatty acids, which could have satiety-inducing, anticancer, and cholesterol-lowering qualities [57], the host’s immune system, and its capacity to combat viruses and disorders are preserved thanks to immunomodulatory actions that are involved in their development [58].

**Figure 2:**
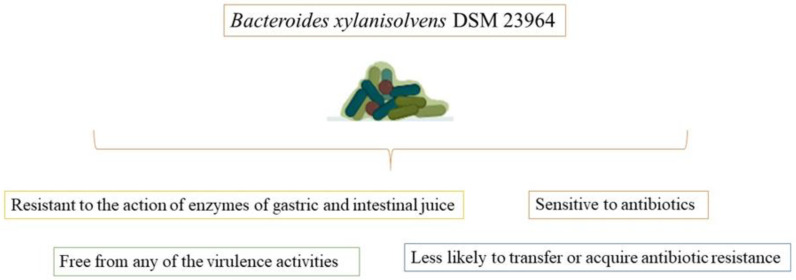
Potential promising advantages of *Bacteroides xylanisolvens* DSM 23964 strain in food fermentation.

*Bacteroides xylanisolvens* DSM 23964 is a novel species of nonpathogenic *Bacteroides xylanisolvens* that was discovered in the feces of healthy human individuals. *Bacteroides xylanisolvens* DSM 23964 strain is free of any virulence factors and is sensitive to antibiotics. It is resistant to the action of the stomach and intestinal juice enzymes during nutrient fermentation (Figure 2). However, additional in vitro and in vivo tests are still required for a thorough safety analysis [59].

The host is sensitive to the defense system, according to the DSM 23,964 strain of *Bacteroides xylanisolvens*, which does not have any virulence factors, carry any mutagenic activity, exhibit any toxicological effects in live or pasteurized form, or exhibit any pathogenic properties in vivo at concentrations up to 3.3 × 10^12^ pasteurized bacteria/kg body weight. This demonstrates that *Bacteroides xylanisolvens* DSM 23964, in both live and pasteurized forms, is safe for use in food [60].

An anaerobic, spore-free, nonmotile, Gram-negative bacteria, *Bacteroides xylanisolvens* DSM 23964 strain does not move short-rod or rod-shaped cells, which are typically 0.4 to 0.5 m broad and 1 to 2 m long. Wilkins-Chalgren agar colonies have a convex surface, are milky, round, elevated, and 2 to 3 mm in diameter [59].

Simulated gastric and intestinal secretions were used to subject the bacteria in the digestive tract to their effects. More than 90.0% of the *Bacteroides xylanisolvens* DSM 23964 strain survived in gastric juice after 180 min, and more than 96% after 240 min of exposure to intestinal juice [59]. The fermentation and breakdown of xylan and other plant fibers are significantly aided by the bacteria *Bacteroides xylanisolvens* [55]. In the human diet, polysaccharides play a significant role in sustaining intestinal commensals such as *Bacteroides xylanisolvens*. They are capable of being converted into short- and branched-chain fatty acids, which are reabsorbed from the large intestine and supply a sizable number of the host’s daily energy requirements [61]. According to data, *Bacteroides xylanisolvens* DSM 23964 is a candidate for probiotics and does not have any virulence characteristics that could impede its usage for future safety and health-promoting evaluation [59].

As a novel food under the Novel Food Regulation No. 258/97, pasteurized dairy products fermented with *Bacteroides xylanisolvens* DSM 23964 received The European Food Safety Authority’s (EFSA) approval in 2015. It is prohibited to use this particular strain as a starter culture in the fermentation of pasteurized dairy products. However, only *Bacteroides xylanisolvens* inactivated cells that have been heat-inactivated were permitted in the final products. Moreover, the EFSA Panel accepted that the procedures followed were industry standards for the dairy sector, that they were sufficiently specified, and that there were no safety issues [62].

Anaerobic bacteria are the predominant forms in the human gut. The ability of a bacterial strain to survive through the digestive system and ultimately provide advantageous effects for the host is a crucial component of its ability to perform nutrient fermentation. Promisingly, after three hours in simulated gastric juice, *Bacteroides xylanisolvens* DSM 23964 showed a 90.0% survival rate, and after four hours in simulated intestinal juice, it had a 96.0% survival rate [59]. By lowering cholesterol levels, inducing satiety, and even having an anticarcinogenic impact, *Bacteroides xylanisolvens*’ synthesis of short-chain fatty acids and the fermentation of dietary polysaccharides are linked to benefits for human health [57].

### 2.3. Akkermansia municiphila

Akkermansia genus member *Akkermansia municiphila* is a microorganism that has recently come to the fore with possible health effects and currently has no history of commercial use in the food industry [29]. However, some experimental data on the production of innovative foods have begun to report promising results on the use of this bacteria, especially in terms of fermentation and probiotic-potential-mediated health effects.

Derrien, Vaughan, Plugge, and De Vos (2004) discovered *Akkermansia muciniphila*, a novel genus of the phylum *Verrucomicrobia* and a member of the commensal gut microbiota. The ATCC BAA-835-type strain of *Akkermansia muciniphila* is the most researched variety [63]. The *Akkermansia muciniphila* genome stands apart from other *Verrucomicrobia* genomes because 28.8% of its genes are shared with the organism’s closest relatives [64]. Using fluorescence in situ hybridization (FISH) and quantitative PCR (qPCR), it was discovered that *Akkermansia muciniphila* accounted for more than 1.0% of the entire microbiota (3.0–5.0% of the gut microbiota in healthy individuals) [65,66]. *Akkermansia muciniphila* generally colonizes the intestinal mucus layer, intestinal colonization is complete at an early age and reaches the level observed in adults within a year. *Akkermansia muciniphila* colonization decreases with increasing age [67]. *Akkermansia muciniphila* is nonmotile, non-spore-forming, oval-shaped, chemo-organotrophic, and it requires mucin as a carbon source along with nitrogen and enzymes that break down mucin [29].

The primary component of mucus, mucin, is a collection of glycoproteins found in mucus discharges. The oligosaccharides N-acetyl-D-galactosamine (GalNAc), N-acetyl-D-glucosamine (GlcNAc), D-galactose L-fucose, and other amino and monosaccharide sugars are glycosylated to produce the mucus layer. The selective permeability provided by this layer enables the movement of nutrients into epithelial cells. The mucus layer offers a surface layer for bacteria to grow and penetrate and is the first line of defense against mechanical harm, pathogens, and toxins [68]. Sulfatases, β-galactosidases, exo-α-sialidases, α and β acetyl-glucosaminidases, neuraminidases, L-fucosidase, and aspartic protease are some of the mucin-degrading enzymes found in *Akkermansia muciniphila*. By decomposing mucin with these enzymes, *Akkermansia muciniphila* generates carbon, nitrogen, and energy sources for the organism or other gut microbiota inhabitants [69,70,71,72,73]. *Akkermansia muciniphila* also degrades mucin and generates short-chain fatty acids such as acetate, propionate, butyrate, and 1,2-propanediol (which is then metabolized to propionate). Additionally, the inflammatory toxicity of sulfate in the mucin layer might be reduced by *Akkermansia muciniphila* employing hydrogen sulfide for cysteine synthesis [74]. By binding to and subsequently activating the signaling pathway to control glucose and lipid metabolism in the peripheral organs, butyrate Gpr41 or Gpr43 are generated by *Akkermansia muciniphila* [66]. Fucose, galactose, N-acetylglucosamine, N-acetylgalactosamine, sialic acid, disaccharide, and tiny oligosaccharides are also released because of mucin degradation. Mucin degradation provides the energy needs of the microbiota and gives an advantage for starvation, malnutrition, and total parenteral nutrition [75]. *Akkermansia muciniphila* increases the activity of L cells and stimulates the release of glucagon-like peptide-1 (GLP-1) and glucagon-like peptide-2 (GLP-2) from L cells. The organism *Akkermansia muciniphila* enhances the release of glucagon-like peptide-1 (GLP-1) and glucagon-like peptide-2 (GLP-2) from L cells and boosts the activity of L cells. In addition to increasing the number of goblet cells and the expression of tight-junction proteins such as zonulin (ZO-1), ZO-2, and ZO-3, *Akkermansia muciniphila* restores the host’s mucus layer thickness to normal [76]. By inhibiting the transfer of lipopolysaccharide (LPS) from the colon to the blood, *Akkermansia muciniphila* lowers endotoxemia and improves intestinal permeability [77]. The *Akkermansia muciniphila* outer membrane protein, Amuc_1100, is essential for contact with the host. The anti-inflammatory and antitumorigenic properties of the Amuc 1100 protein, the restoration of tryptophan levels, and the stimulation of serotonin metabolism all impact the health of the host [78]. Additionally, the toll-like receptors (TLR2) signaling pathway allows the particular cytokine IL-10 to be produced when the *Akkermansia muciniphila* outer membrane protein Amuc 1100 is present [79].

**Figure 3:**
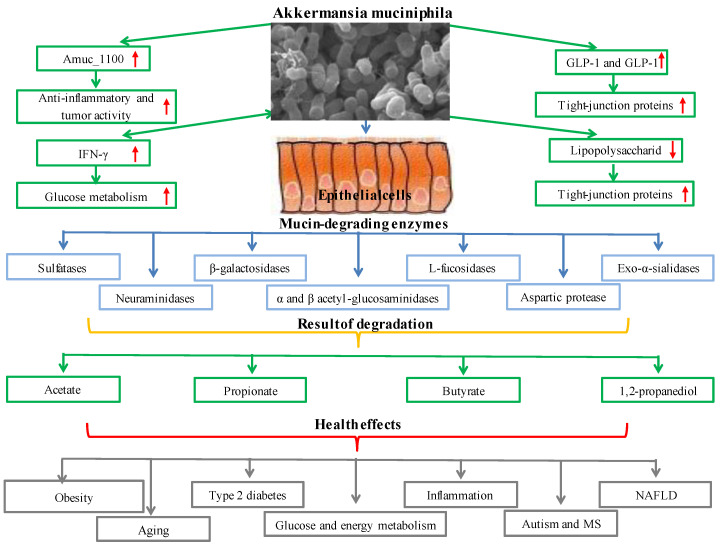
Metabolic functions and health effects of *Akkermansia muciniphila*.

Systemic glucose metabolism is impacted by IFN-γ a key immune system cytokine [80]. IFN-γ regulates the production of genes such as the immune-associated GTPase family (Irgm1), guanylate-binding protein 4 (Gbp4), and ubiquitin D(Ubd), which helps control the amount of *Akkermansia muciniphila* in the gut. *Akkermansia muciniphila* mediates the impact of IFN-γ on glucose tolerance through this pathway [81,82]. The effects of *Akkermansia muciniphila* include reducing metabolic inflammation, enhancing intestinal integrity, boosting intestinal peptide hormone secretion, and improving metabolic parameters. Because of these effects, *Akkermansia muciniphila* is one of the most promising biotherapeutic agents for metabolic diseases, including obesity ([83], type 2 diabetes [84], inflammation, glucose and energy metabolism [85], nonalcoholic fatty liver disease [86], aging, autism, and multiple sclerosis [87,88,89]. It has been determined time and time again that *Akkermansia muciniphila* is a crucial part of the gut microbiota [90,91,92]. The functional and metabolic functions and health effects of *Akkermansia muciniphila* are presented in Figure 3.

### 2.4. Mycobacterium setense manresensis

*Mycobacterium setense manresensis* is a microorganism that has stood out recently, especially in terms of novel food production; it is an encapsulated ingredient composed of ≤10^5^ heat-killed, freeze-dried *Mycobacterium setense manresensis* [93]. Recently, some opinions have begun to be put forward regarding the use of this microorganism in fermentation and probiotic production although there is limited information in the literature.

**Figure 4:**
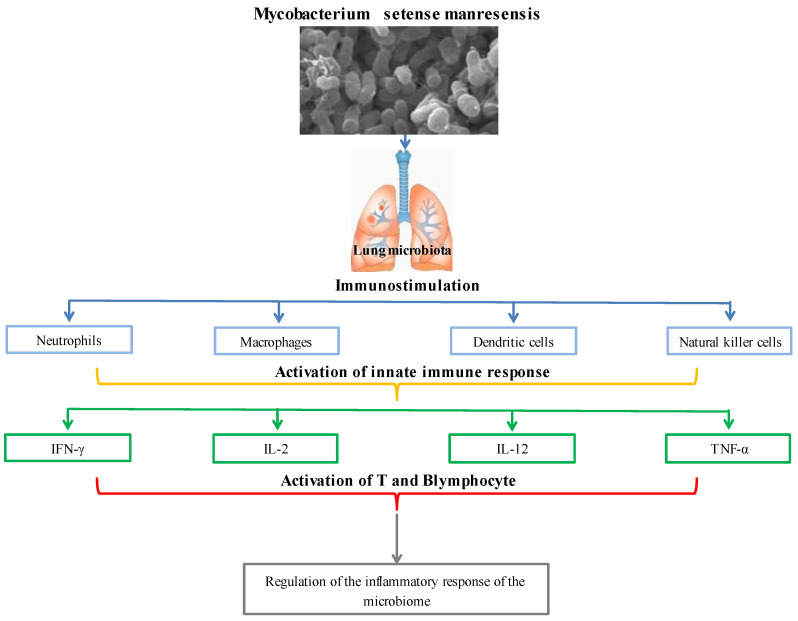
Immune system regulation of *Mycobacterium setense manresensis*.

Quickly proliferating and commonly recognized nontuberculous nonpathogenic mycobacteria (NTM) species known as *Mycobacterium fortuitum* cause localized skin and soft tissue infections. Numerous strains of the *Mycobacterium fortuitum* complex are also known as *Mycobacterium peregrinums*, *Mycobacterium porcinum*, *Mycobacterium septicum*, *Mycobacterium conceptionense*, *Mycobacterium boenickei*, *Mycobacterium houstonense*, *Mycobacterium neworleansense*, *Mycobacterium brisbanense*, *Mycobacterium farcinogenes*, and *Mycobacterium senegalense* [94,95]. With their adaptable ecological and symbiotic biological characteristics, nontuberculous nonpathogenic mycobacteria may thrive in various habitats, from harsh natural surroundings to microniches in the human body [96]. Nontuberculous and nonpathogenic mycobacteria stimulate the local lung microbiota, neutrophils, macrophages, dendritic, and natural killer (NK) cells to activate the innate immune system. Toll-like receptors (TLRs) and nod-like receptors in the activated innate immune system allow for the identification of mycobacterial and microbial pathogen-associated molecular models (PAMPs) (NLRs). Recognized PAMPs control the microbiome’s inflammatory response by activating T and B cells, primarily through interactions with interferon-γ (IFN-γ), interleukin (IL)-2, IL-12, and TNF-α [97]. TRAF6, an essential signaling molecule in TLR-triggered inflammation, is deubiquitinated by the anti-inflammatory protein A20 because of *Mycobacterium fortuitum* induction. By increasing TNFAIP3, which blocks TNF-induced signaling, and by blocking both MyD88-dependent and -independent TLR-induced NF-Kβ pathways, the A20 enzyme lowers inflammation. It has been claimed that *Mycobacterium fortuitum* A20 expression controls the host’s proinflammatory responses negatively [98]. *Mycobacterium setense*, a brand-new species that is a member of the *Mycobacterium fortuitum* complex, was discovered in France in a patient who was 52 years old and had soft tissue infection and osteitis. The nonpathogenic group of nontuberculous mycobacteria includes it [99,100]. This novel strain was given the name *Mycobacterium setense manresensis* and shares characteristics with *Mycobacterium setense* and other genes frequently used to identify *Mycobacterium* species, including AsrpoB, rpoC, hsp65, and sodA. In Catalonia, Spain, a nonpathogenic strain of *Mycobacterium setense manresensis* was found on a riverbank. The 6.06 Mb *Mycobacterium setense manresensis* genome had 22 contigs with an average coverage depth of 788. A similar *Mycobacterium* species GC content was found in the Manresensis strain (66.5%) [101]. Drinking water contained a new species of *Mycobacterium setense manresensis*, a member of the *Mycobacterium fortuitum complex* (which also includes nontuberculous bacilli responsible for skin, lymph nodes, and joint infections) [102]. Probiotics promote mucosal response and barrier and epithelium repair activities with the SCFAs they produce. They also stimulate IgA to raise IL-10 levels and induce CD4^+^ Foxp3^+^ T-reg by blocking the generation of proinflammatory cytokines. It can interact with mucosal epithelium and the resident cells of innate and adaptive immunity, modulating the host’s local and systemic mucosal immune response [102]. Additionally, it controls the immune response’s regulatory mechanisms by activating TLR2 and TLR4, enhancing NK cell activity and IFN-γ production by producing IL-12, and deactivating T-regs with the anti-inflammatory cytokine Th17 [103,104]. The immune system regulation of *Mycobacterium setense manresensis* is presented in Figure 4. *Mycobacterium setense manresensis*, a novel species from the fortuitum group discovered in drinking water, was given orally for two weeks during a typical tuberculosis treatment. This treatment both eradicated the bacilli and had an excessive impact on the patient’s condition. It was highlighted that it promoted a balanced immune response that placed a strong emphasis on managing the inflammatory response [105]. Total adenosine deaminase, haptoglobin, local pulmonary chemokine (C-X-C motif) ligands-1 and 5, TNF-a, IL-1b, IL-6, and IL-10 are all decreased by taking *Mycobacterium setense manresensis* orally [106]. *Mycobacterium manresensis* is present in Nyaditum resale^®^, one of the probiotics which is a galenic preparation of heat-killed *Mycobacterium manresensis* (hkMn). Preclinical investigations using the strain C3HeB/FeJ of murine active tuberculosis have demonstrated that daily treatment of NR containing 103–106 hkMn for 14 days can halt the development of active tuberculosis. After 7 days of ex vivo treatment of splenocytes with tuberculin-purified protein derivative (PPD) memory-specific Tregs (CD39^+^ CD25^+^ CD4^+^ cells), the administration of low-dose Nyaditum resale^®^ was linked to an increase in these cells. The development of tuberculosis was inhibited by this increase in Tregs, which was also accompanied by an increase in IL-10 in the spleen and a decrease in IL-17 in the lungs [107]. As a result, the lesions’ development and neutrophilic infiltration were paused, which was expected to provide the lesions enough time to encapsulate [108]. In human randomized, double-blind, placebo-controlled clinical trials, Nyaditum resale^®^ significantly increased the number of memory regulatory T cells with specificity for PPD [107].

### 2.5. Novel Lactic Acid Bacteria (Fructophilic Lactic Acid Bacteria (FLAB))

**Figure 5:**
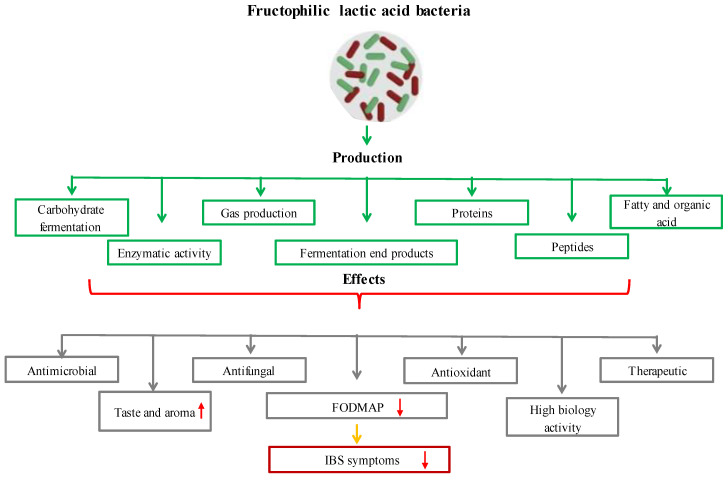
The effects of functional and metabolic properties of FLAB on health and food production.

Recent research has revealed a brand-new breed of *LAB* known as *fructophilic lactic acid bacteria (FLAB)*, which prefer fructose to glucose as a growth substrate [109]. *FLAB* is found in fructose-rich niches, which are the climatic and biological circumstances in which a species should survive, develop, and procreate [110]. Most *FLAB* grow best at pH 5–6 and temperatures of 30–35 °C [111]. *FLAB* are capable of carbohydrate fermentation (fermenting hexoses and pentoses), enzymatic activity, and gas, fermentation end products, proteins, peptides, oil and organic acid production. *FLAB* can have antimicrobial properties [111,112,113,114,115]. Due to the absence of the adhE gene, which codes for alcohol/acetaldehyde dehydrogenase, *FLAB* are heterofermentative *LAB*-type microbes that additionally create acetic acid and trace amounts of ethanol (ethanol, lactic acid, acetic acid = ratio 1:1:0.2, and mannitol) [116]. The plant secondary metabolite *p*-coumaric acid, which is a structural component of sporopollenin, the primary matrix that creates the exterior of pollen grains, is produced by *FLAB* using these [117,118]. *FLAB* have enzymes that can convert *p*-coumaric acid to 4-vinylphenol in the first step and 4-ethylphenol in the second stage [119]. These secondary metabolites are biologically active and have significant antioxidant capacities; they may also enhance the flavor of fermented foods [120]. At present, *FLAB* consist of two genera, *Fructobacillus* and *Lactobacillus*, and include six species, *Fructobacillus durionis, Fructobacillus fructosus*, *Fructobacillus pseudoficulneus*, *Fructobacillus tropaeoli*, *Lactobacillus kunkeei*, and *Fructobacillus ficulneus*, classified by Endo as obligatorily fructophilic, and only one species, namely *Lactobacillus florum*, is facultatively *fructophilic* [121]. *FLAB* are associated with the genera *Leuconostoc*, *Convivina*, *Fructilactobacillus*, *Weissella,* and *Oenococcus* [116]. New species with possible fructophilic characteristics are still being found, though [122]. *FLAB* have recently been discovered in the gastrointestinal tracts of animals that ingest fructose, including bumblebees, tropical fruit flies, and *Camponotus* ants. *FLAB* have previously been discovered in flowers, fruits, and fermented foods made from fruit [123]. Fermentation and *LAB* together give food significant organoleptic, quality, and safety advantages. As a source of water-soluble vitamins, dietary fiber, phytosterols, phytochemicals, and minerals, fermented vegetables (such as cucumber, Korean sauerkraut, capers, carrots, and table olives) are crucial to human nutrition. A new generation of multifunctional-starting cultures can be used to produce products with greater usefulness while also improving quality and safety, reducing economic losses and spoilage, and improving process control [124]. Given that they contain various LAB, certain fermented fruits and vegetables can be employed as a potential source of probiotics. As a whole, traditionally fermented fruits and vegetables may provide health benefits in addition to acting as a dietary supplement [125]. One can divide the *FLAB* into two categories. The first group includes the representatives *Fructobacillus fructosus* and *Lactobacillus kunkeei* as well as the partially related *Lactobacillus apinorum* and *Lactobacillus florum*, which are linked to flowers, grapes, wine, and insects. The second group, which consists of the bacteria *Fructobacillus ficulneuses*, *Fructobacillus pseudoficulneus*, and *Fructobacillus durionis*, is connected to ripe fruit and fruit fermentation (except grapes and wines). Between the two categories can be found *Fructobacillus tropaeoli*, which is present in flowers, fruits, and fruit fermentation. *FLAB* are referred to as promising microorganisms that can improve human health [126]. The evaluation of *FLAB*’s advantageous traits has gained attention due to the possible use of these novel probiotics [111]. *FLAB* strains are mostly obtained from settings high in fructose, such as the honeybee microbiome and bee products (*Lactobacillus kunkeei* and *Fructobacillus fructosus*). There is only one report of the isolation and identification of the relatively new *FLAB* strain *Lactobacillus apinorum* in bees [126]. In this investigation, samples of pollen and bee bread were used to isolate 27 distinct strains of four *FLAB* species. *FLAB* strains displayed high levels of autoaggregation and hydrophobicity in terms of functional characteristics. Importantly, it was discovered that the strains of *Lactobacillus kunkeei* and *Fructobacillus fructosus* had low levels of bile salt output and limited pH tolerance. The significance of *FLAB* strains’ functional roles for upcoming applications is increased by their high levels of antibacterial and antifungal activity [127]. A different study found that specific *Lactobacillus kunkeei* strains had antibacterial effects on honeybee larvae that were afflicted with the foulbrood disease *Melissococcus plutonius* [128,129]. Another study found that *FLAB* played a significant role in honey production by bees and were abundant in fresh honey. These bacteria reside in the microbiome of honeybees. It was also noted that fresh honey would soon be the best alternative for wound healing due to the antibacterial and therapeutic qualities of *FLAB* [130]. Irritable bowel syndrome (IBS) and other functional bowel diseases have been linked to the consumption of fermentable oligosaccharides, disaccharides, monosaccharides, and polyols (FODMAP). A study indicated that by fermenting wheat dough, *FLAB* significantly lowered the number of FODMAPs present in it [131]. Wine flavor and aroma can be improved by *Lactobacillus florum*, which produces the genes for citrate lyase, phenolic acid decarboxylase, and malolactic enzyme [132]. Another study found that the fermentation of plant meals by *Lactobacillus florum* 2F resulted in the production of two polyols, erythritol and mannitol [133]. *Fructobacillus durionis* was found in tempoyak, a fermented condiment made from the pulp of durian [134]. The formation of flavor and aroma was influenced by the fermentation of cocoa beans by the bacteria *Fructobacillus durionis*, *Fructobacillus pseudoficulneus*, *Fructobacillus ficulneus*, and *Fructobacillus tropaeoli* [135]. In another study, the *Fructobacillus tropaeoli* CRL 2034 strain was used to create mannitol alcohol, which has a zero glycemic index and is used in diets for diabetes. As a consequence, 81.91 g/L of mannitol with a 77.47% yield was produced by *Fructobacillus tropaeoli* CRL 2034 [136]. The fermentation of cocoa beans by *Lactobacillus plantarum* LPBF 35 resulted in the production of many aroma-active compounds, including acetaldehyde, ethyl acetate, nonanal, and octanoic acid, as well as an ideal organic acid metabolism profile, which included the consumption of both lactic acid and citric acid [137,138]. Recent studies have revealed that taking *Lactobacillus kunkeei* as a probiotic may have positive effects on human health, including increased bowel movements and improved immunoglobulin A production [139]. As a result, almost a century after the genus *Lactobacillus* was initially described, scientists are attempting to develop many biotechnological and medicinal advances. The ecological idea included in Pasteur’s adage, “never underestimate the power of the microbe”, is clearly reflected by the finding of *FLAB* and their fructophilic metabolic capabilities. This is particularly true in this case [140]. The effects of the functional and metabolic properties of *FLAB* on health and food production are presented in Figure 5. Since ancient times, *LAB* have been involved in the creation of human food (fermentation) and nutrition and have usually been regarded as safe [141]. Recent research has identified the existence of *FLAB* in several foods [142,143]. Although the *fructophilic lactic acid bacterium Apilactobacillus kunkeei*’s postbiotic qualities have only been studied in human clinical trials [144], its possible probiotic properties have only been investigated in in vitro studies [145]. In a study, *Apilactobacillus kunkeei’s* existence of helpful enzymes (β-glucosidase, β-galactosidase, and leucine arylamidase) revealed many intriguing characteristics, including minimal antibiotic resistance and the capacity to inhibit. The study also demonstrated that *Apilactobacillus kunkeei* could be used for probiotic action in fruit-based diets, which are frequently consumed by hospitalized and immunocompromised patients [144]. The limited adoption of *FLAB* as probiotics was caused, in part, by the lack of information on the eating patterns of organisms that harbor living things [146].

### 2.6. Some Other Novel Microorganisms

Newly discovered genera or species from the natural world or bacterial strains created from pre-existing bacteria are both examples of novel microorganisms. These microorganisms have potential applications in the preservation of food, textural modification, and gut microbiota modulation [29]. Among these new-generation microorganisms are *Faecalibacterium prausnitzii* [147], *Lactobacillus rhamnosus* R0011 (dairy fermentation and functional juice production), *Lactobacillus helveticus* R0052 (dairy fermentation) [148], *Lactobacillus acidophilus*, *amylovorus*, *casei*, *gasseri*, *johnsoniii*, *pentosus*, *plantarum*, *reuteri*, *Bifidobacterium bifidum*, *breve*, *infantis*, *longum* (dairy fermentation), *Enterococcus faecium* (cheese and sausage production), *Lactococcus lactis* (dairy fermentation and sauerkraut production), *Streptococcus* (dairy fermentation) [149], *Leuconostoc mesenteroides* (carbohydrate fermentation), *Bacillus subtilis* Natto (soy fermentation) [29], *Saccharomyces cerevisiae (boulardii)* (production of bakery products) [150], *Corynebacterium glutamicum* (amino acid production) [151], *Torulaspora delbrueckii* JK08 (production of bakery products), *Pichia anomala* (dairy fermentation and production of bakery products) [152], and *Propionibacterium freudenreichii* (dairy fermentation) [153].

To sum up, microorganisms are typically employed in the production of dairy products, some fermented foods (traditional olives, pickles, sauerkraut, etc.), fermented meats, sourdough breads, etc. Additionally, for the manufacturing of wine and other alcohol, chocolate, pigments and their use in the preservation of fruits, vegetables, and meat, they began to be employed widely as probiotics known as helpful to human health [154]. Today’s quick technological advancements and applications reveal unique microorganisms’ positive qualities. These innovations enhance the health-promoting abilities of new microorganisms [155].

### 2.7. The Safety Issues of Novel Microorganisms

Novel microbes are either newly isolated genera and species from natural sources or strains of bacteria derived from already existing bacteria. Novel microbes are gaining increasing interest for the general purposes of food preservation and modification and gut microbiota modulation [156]. The use of novel microbes to improve health outcomes, despite the need for a thorough evaluation of their safety [29]. Each novel bacterium differs in its safety and approval status and forms by authoritative institutions. For *C. butyricum*, the European Commission approved the use of *C. butyricum* CBM 588 as a novel food ingredient in 2014, and then some companies in the UK began to evaluate its use in food supplements [29]. Although the demands for its use are mostly related to its positive effects on lipid metabolism and gut microbiome modulation, this bacterium has many promising potentials in terms of food, the environment, and health [30,31]. On the other hand, *Bacteroide xylanisolvens* DSM 23964, a bacterium with a short-term history, has been used in the production of a novel food by taking place in a heat-treated, nonviable form during the fermentation process of a heat-treated (pasteurized) dairy product and has attracted attention with the acceptance of the European Commission [29].

Furthermore, *Akkermansia muciniphila* has been discovered to have positive results and these effects intensify rather than diminish after pasteurization. After pasteurization, it was suggested to use *Akkermansia muciniphila* as a food supplement. No negative effects were seen when *Akkermansia muciniphila* was given orally for 90 consecutive days at doses of 75, 375, or 1500 mg/kg body weight/day (4.8 × 109, 2.4 × 1010 or 9.6 × 1010) and no adverse effects were observed [157]. According to another study, people who consumed natural yoghurt had significantly greater concentrations of *Akkermansia muciniphila* in their feces [158]. Another study found that combining fructooligosaccharides with *Akkermansia muciniphila* boosted the production of butyric acid in the digestive tract by 32% [159]. In one study, the microbiota colonization of *Akkermansia muciniphila* and the digestion of human milk oligosaccharides were examined. As a result, *Akkermansia muciniphila* could ferment human milk and colonize the mucosal layer early in life, improving mucosal and metabolic health warranting later life [71]. The EFSA Panel on Nutrition, Novel Foods and Food Allergens (NDA) was invited to state their position on pasteurized *Akkermansia muciniphila* as a novel food (NF) in conformity with Regulation (EU) No 2015/2283 at the request of the European Commission. A well-characterized, non-toxin-producing, and avirulent bacteria called *Akkermansia muciniphila* has been identified as a typical component of the gut microbiota [160]. As a result, *Akkermansia muciniphila* may provide helpful metabolites to promote the gut microbial balance. No reports of individual pathogenicity have been reported up to this point. It is unknown if *Akkermansia muciniphila* causes disease in conjunction with other bacteria. However, more extensive clinical research is required to guarantee the safety of *Akkermansia muciniphila* use in the future [87].

For *Mycobacterium setense manresensis,* the EFSA Panel on Nutrition, Novel Foods, and Food Allergens has determined that heat-killed *Mycobacterium setense manresensis* is a novel food under EU Regulation 2015/2283 and can be used for the general adult population excluding children, pregnant, and lactating women [161]. On the other hand, promisingly, *FLAB’s* lack of clinical evidence of toxicity may be a sign of possible safety. However, due to the lack of knowledge on how consumers are exposed to this particular kind of bacteria through food, this possibility should be viewed with caution [29]. EFSA and the Food and Drug Administration (FDA) do not identify *FLAB* as having qualified presumptions of safety or as generally recognized as safe for now [148]. Recent studies have revealed that taking heat-killed *Lactobacillus kunkeei* YB38 may have health benefits for people, including more frequent bowel movements and a greater production of immunoglobulin A [153,162]. Consequently, it is believed that *Lactobacillus kunkeei among FLAB* may be part of the food industry [162].

**Figure 6:**
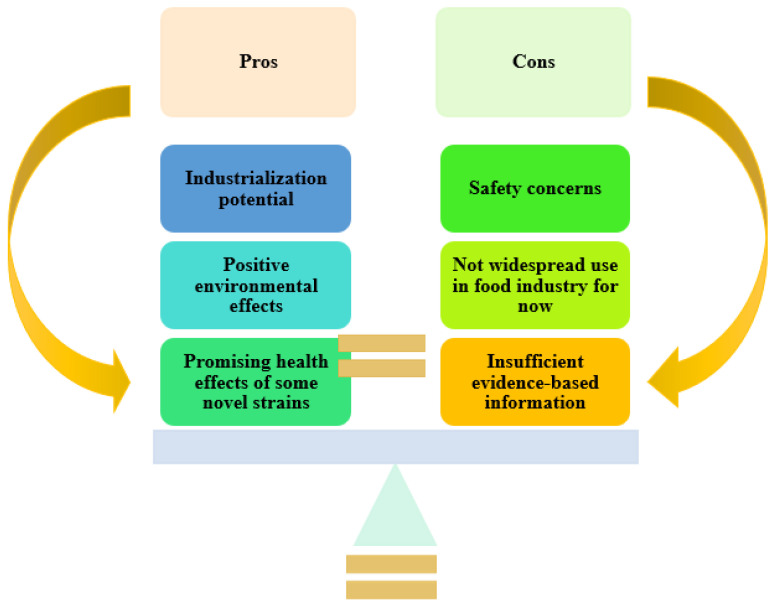
Advantages and disadvantages of novel microorganisms compared to traditional fermentative ones.

In conclusion, the potential health benefits and applications of novel microorganisms, which are thought to enter the industrialization process rapidly, are expanding daily because they have some advantages in different areas, but there are also usage concerns as the most important disadvantages. Figure 6 compares some pros and cons of these novel bacteria compared to traditional ones. Whether the advantages outweigh the disadvantages will undoubtedly be determined by time.

## 3. Conclusions

Fermentation technology is one of the technological processes that both the food industry and the scientific world are most interested in because of their functional effects on food and health. Microorganisms play a crucial role in food production, processing, preservation, and storage, making them a vital component of the food industry and health. In this context, the potential for use of both old- and new-generation microorganisms in the food industry, especially in fermentation technology, comes to the fore. LAB are generally considered to be safe and have been involved in different food products. Newly discovered genera or species from the natural world or bacterial strains created from pre-existing bacteria are both examples of novel microorganisms and based on the knowledge acquired so far, some novel microorganisms can be very interesting for the food industry in terms of their effects on food, health, and the environment.

When it comes to future perspectives and challenges of new-generation microorganisms, it is thought that these microorganisms, which have started to be integrated in food products and get approved, will quickly take place in food fermentation technology. However, the most important challenges here are, of course, the safety issues of certain novel microorganisms, the cost and standardizations of obtaining them, and the fact that their effects on the functionality of the food products are not yet known. These novel microorganisms can have a synergistic effect with traditional ones, and may also have negative effects, as well. For these reasons, they have not yet been industrialized, especially in the food industry, as much as in the functional food market.

In conclusion, although some novel microorganisms have promising properties for food fermentation and especially health, comprehensive research is still needed to better understand these microorganisms, particularly regarding their functionality for fermentation especially food fermentation and safety issues.

## Data Availability

Data is contained within the article.
